# Biomimetic Scaffolds in Skeletal Muscle Regeneration

**DOI:** 10.15190/d.2019.3

**Published:** 2019-03-31

**Authors:** Greta D. Mulbauer, Howard W.T. Matthew

**Affiliations:** Department of Chemical Engineering and Materials Science, Wayne State University, Detroit, MI, USA

**Keywords:** Tissue engineering, regenerative medicine, artificial muscle, biomaterials, biomimetic scaffolds, volumetric muscle loss.

## Abstract

Skeletal muscle tissue has inherent capacity for regeneration in response to minor injuries. However, in the case of severe trauma, tumor ablations, or in congenital muscle defects, these myopathies can cause irreversible loss of muscle mass and function, a condition referred to as volumetric muscle loss (VML). The natural muscle repair mechanisms are overwhelmed, prompting the search for new muscle regenerative strategies, such as using biomaterials that can provide regenerative signals to either transplanted or host muscle cells. Recent studies involve the use of suitable biomaterials which may be utilized as a template to guide tissue reorganization and ultimately provide optimum micro-environmental conditions to cells. These strategies range from approaches that utilize biomaterials alone to those that combine materials with exogenous growth factors, and ex vivo cultured cells. A number of scaffold materials have been used in the development of grafts to treat VML. In this brief review, we outline the natural skeletal regeneration process, available treatments used in the clinic for muscle injury and promising tissue bioengineering and regenerative approaches for muscle loss treatment.

## **SUMMARY**

**1. Introduction**

**2. Natural Skeletal Muscle Regeneration**

*2.1 Inflammatory phase*

*2.2. Repair phase*

*2.3. Remodeling phase*

**3. Current Treatments**

*3.1 Surgery*

*3.2 Physical therapy*

*3.3 Scaffolds and cell-based therap*y

*3.4 Other strategies*

**4. Promising Tissue Engineering Treatments**

*4.1 Acellular and cellular scaffold-based strategies*

*4.2 Cell-based strategies*

*4.3 Other strategies*

*4.4 Challenges and limitations*

**5. Towards a Human Host: Bioscaffolds and Cell-therapy in Clinical Trials**

**6. Conclusion**

## **1. Introduction**

In the human body, skeletal muscle tissue is the most abundant tissue type, totaling 40-45% of the body weight. Skeletal muscle plays an essential role in body movement through contraction and relaxation, postural support, temperature regulation and breathing^1,2^. Its robust innate regenerative ability requires a constant flow of nutrients and metabolites, which it receives from a complex capillary network forming a three-dimensional pattern throughout the muscle fibers^3^. Under circumstances of severe injuries – muscle mass loss greater than 20%^1^ – tissue repair response is incapable of adequate tissue regeneration, and may lead to extensive and irreversible fibrosis, scarring, and loss of muscle function^4^.

Major trauma with lasting functional impairment sustained from motor vehicle accidents, combat- or sport-related injuries, aggressive tumor ablation, prolonged denervation and other causes is defined as volumetric muscle loss (VML)^4-7^. VML significantly impacts patient’s movement ability^5^. VML can result from progressive muscle loss, as found in inherited genetic disorders with or without metabolic implications, such as Amyotrophic Lateral Sclerosis, Charcot-Marie Tooth disease and Duchenne Muscular Dystrophy^8-10^. VML can also result from nerve damage (leading to muscle atrophy), diabetes, heart diseases (heart failure) and chronic kidney diseases^5,11,12^.

While for non-severe muscle loss there are several non-surgical related treatment strategies available, such as RICE (combination of rest, ice, compression and elevation)^1,13^, in VML, specific surgery-based strategies are usually employed^14^. Namely, the standard treatment in such cases is the autologous transplant from an uninjured site, by collecting healthy muscle tissue from a normal site and transferring it into the injured site, after the damaged muscle tissue is removed^15^. Notably, about 1 in 10 of all these surgical procedures result in complete graft rejection, due to cell death (necrosis) and potential infections^15^. These surgical strategies always lead to a reduced muscular function, due to the subsequent fibrosis and scarring of the tissue, even when the surgery is a success^16^. Moreover, injuries that are extended on a larger portion of the limb may result in amputation of that limb. Thus, there is an acute need for additional strategies to improve muscle loss recovery. Such strategies can include**complex muscle structures for implantation and replacement of the missing muscles, or tissue-like scaffolds that can provide regenerative signals to either transplanted or host muscle cells, in order to enhance skeletal muscle recovery and regeneration^1,5,14^.

## **2. Natural Skeletal Muscle Regeneration**

Adult skeletal muscle has an extraordinary capacity to regenerate and repair after an injury, despite the fact that it is a stable tissue in regular conditions. Skeletal muscle regeneration is a complex and highly regulated process involving the activation and interplay of various processes, such as the inflammatory response, growth factor and survival signaling, stem cells-mediated repair regeneration of muscle cells and fibroblast infiltration with or without scar tissue formation^2^.**Skeletal muscle regeneration can be classified in three phases, that partially overlap: Inflammatory/Destruction phase, Repair phase and Remodeling phase^1^.

The major limitations in VML injury repair of the affected muscle are the complete destruction/removal of the basal lamina and the loss of other structural muscle constituents, such as the damage to the stem cell niche (e.g. satellite cells)^2^.

### *2.1 Inflammatory phase*

Inflammatory phase, also called the Destruction phase, is characterized by the initial acute injury resulting in significant damage of the myofibrils and surrounding tissue with necrosis^17,18^. The main initial event of the Inflammatory phase is the recruitment of the neutrophil granulocytes, innate immune cells with anti-bacterial activity, cells that migrate to the injury location from the bloodstream. Neutrophils have the role of cleaning the injury site from generated debris and to remove significantly damaged myofibrils. Neutrophils can also promote vascularization, a VEGF-A-mediated process. However, neutrophils may also produce cytotoxic compounds which may affect skeletal muscle regeneration^3^. Neutrophil infiltration event peaks at 2 hours post-injury. Other immune cells, such as eosinophils, can migrate to the injury site and activate the fibro-adipogenic precursor (FAP) cells (CD45-, CD31-, α7-Integrin- and CD23+, Sca-1+) from the interstitial space. These initial processes are followed by migration of the macrophages^19^.

Macrophages are specialized phagocytes that take over the lead as the main cell type at destruction site in about 2 days^19^. Initially, pro-inflammatory M1 macrophages (CD68+CD163-) help in phagocytosis of remaining necrotic myofibers and in supporting the stem cells by promoting their survival and proliferation^20^, with a peak at about 24 hours post-injury. After ~3 days, anti-inflammatory M2 macrophages (CD68-CD163+) replace M1 macrophages and enable muscle tissue regeneration by promoting myoblast proliferation and function. The main trigger of this M1 to M2 switch (inflammatory to anti-inflammatory phenotype) is represented by the phagocytosis of necrotic and/or apoptotic myofibers and other damage tissular components. Different studies suggest the involvement of the IL-10 secretion in M1 to M2 switch^21,22^. Notably, if M1 cells persist, their secreted cytokines (e.g. IFN-gamma) may inhibit the M2 phenotype differentiation and recruitment^23^. The bridge between the Inflammatory phase and the Repair phase is represented by the interaction between the macrophages and the stem cells^3^.

### *2.2. Repair phase*

**Figure 1:**
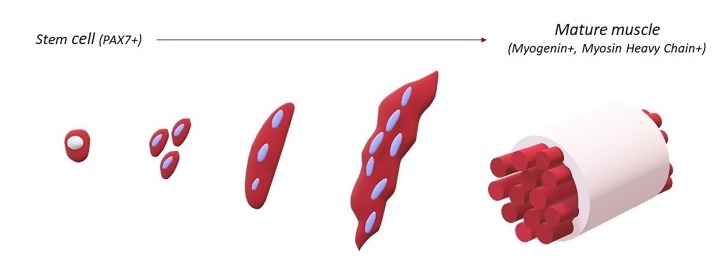
Stem Cells in Muscle Regeneration Stem cells infiltrate the injured area, transform into myoblasts, which fuse and further group into muscle fibers, leading to skeletal muscle regeneration.

The major event taking place within the Repair phase is the activation of the stem-cell niche, which is in part promoted by the macrophages. Satellite cells, as the skeletal muscle stem cells, play a major role in formation of new muscular cells and reassembling of the contractile apparatus^2^. Once the injured tissue has cleared its debris, stem cells infiltrate the injured area, where they later transform into myoblast, which fuse and further group into muscle fibers^3^ **(Figure 1**). Noteworthy, only a part of the stem cells is able to proliferate and differentiate into a different phenotype^3^.

Initial activation of stem cells is mediated by the M1 macrophages and their secreted cytokines, such as the TNF-alpha, which stimulates the division of stem cells. M2 macrophages come then into play, promoting the differentiation of the stem cells through the secretion of IL-4 and IL-10. While quiescent stem cells express several biomarkers, such as Pax-7, M-Cadherin, and CD-34, in the activated stem cells the expression of Pax-7 is reduced and instead they start to express the basic Helix-Loop-Helix (bHLH) transcription factors during their differentiation^24^. Unlike bone injuries, where the bone is repaired by solely generating new bone, an injured muscle instead requires another type of cell – the fibroblast – which is responsible for generating the connective tissue at the site of the injury. It is through this combination of connective tissue with muscle fibers that the injured muscle is repaired. This phase peaks at ~ two weeks and it also involves generation of new blood vessels and nerves^25^.

### *2.3. Remodeling phase*

The final phase in muscle regeneration involves resolution of the initial extracellular matrix, followed by formation of a definitive structure, with the basal lamina playing the role of a regenerative template for the growth of myofibers and neuromuscular junctions^26^. Remodeling phase is dependent upon vascularization and innervation of the healing area, among others. In general, the resolution of the remodeling phase will result in the regeneration of muscle tissue maintaining the initial structure and architecture, or the formation of a scar tissue with separation of myofibers^3^.

Myofibroblasts that are regenerated will fuse with the existing components of the musculature at the injury border^16^. Fibroblasts will infiltrate the injury site (as early as the inflammatory phase) and help regeneration by providing support and replacing the damaged connective tissue^13^. However, while fibroblast infiltration and involvement are generally considered a good event, excessive collagen deposition by the fibroblasts can result in scar tissue formation, which can affect the regenerative process and the muscle functional performance after recovery^1^.

## **3. Current Treatments**

The standard treatment in major muscle injury and loss (VML) is the autologous transplant from an uninjured site, by transferring the healthy segment of muscle into the injured site, after the damaged muscle tissue is removed^15,27^. This is followed by physical therapy. Additional clinically relevant strategies to treat VML involve the use of scaffolds and acupuncture^5^. In contrast, for non-severe muscle loss there are several non-surgical related treatment strategies available, such as the RICE (rest, ice, compression, elevation)^1,13^.

Non-surgical treatment strategies vary based on the skeletal muscle’s regeneration phase. In the *Inflammatory phase***, **application of RICE and/or immobilization for three days to one week may be beneficial in reducing pain and limiting the destruction effects to the injury area^28^. Transition from immobilization to early mobilization is performed within the *Repair phase*. Muscle contraction and stretching stimulates growth of new blood vessels and muscle fibers, while simultaneously reducing formation of the scar tissue and increasing muscle fibers’ tensile strength^13^. Initially, isometric exercises are employed. These are gentle exercises in which one contracts muscle but do not engage movement of the limb or joint. Isometric exercises are then slowly replaced by isotonic exercises, which will strengthen the muscle by enabling its entire range of motion. Under the critical supervision of an expert physical therapist, the isometric and/or isotonic exercises are combined with gentle stretching, which is standard for this phase^28^. In the *Remodeling phase* however, manual therapy together with specialized treatment strategies – Gastron or ASTYM – are employed. These methods will help with the orientation and restriction prevention related to the scar tissue, as well as in decreasing the probability for reinjury. This phase facilitates the return to regular muscle function and regaining of the full strength of the muscle, through isokinetic and sports-specific training^13,28^.

### *3.1 Surgery*

Autologous muscle tissue transplant from an unaffected site to the injured muscle tissue is a standard treatment in major muscle loss situations, such as VML^15,27,29^. In high-level muscle nerve injuries, when adjacent muscle is not available, autologous muscle transfer together with neurorrhaphy, surgical suturing of a ruptured nerve, is employed, as a free functional muscle transfer^5^.

The most common donor muscles for the autologous transfer are the latissimus dorsi muscle and the gracilis muscle^5^ For example, the use of the latissimus dorsi muscle transfer was successfully employed to restore flexion of the elbow after injuries^30^ and to improve versatility in reconstruction of hip abductors^31^, while the gracilis muscle transfer restored the flexion of the elbow after the pan-brachial plexus injury^32^ and was used in microsurgical smile/facial reanimation^33^. In the latter study, sixteen of the patients underwent coaptation of the nerve to the masseter muscle (one-stage reconstruction), while two patients received cross-facial nerve grafts (two-stage reconstruction)^33^. Several other muscles, such as the gastrocnemius muscle in repairing postoperative infection of the patellar internal fixation^34^, and rectus femoris muscle in maxillary reconstruction after maxillary cancer ablation, were also successfully used as donor muscles in autologous transplant^35^.

Notably, about 1 in 10 of all these surgical procedures result in complete graft rejection, due to necrosis and potential infections^15^. Surgical strategies almost always lead to a reduced muscular function, due to the subsequent fibrosis and scarring of the tissue, and inadequate innervation, even when the surgery is a success^5,16^. Sometimes, the source of autologous muscles for grafting may be low or non-existent if the patient is severely injured. Injuries that are extended to a large area of the limb may result in the amputation of that limb. Thus, there is an acute need for novel therapeutic strategies.

### *3.2 Physical therapy*

In VML, exercise and physical therapy are usually used after surgery as a non-invasive strategy to stimulate tissue repair, regeneration and muscle functional recovery. Physical therapy is not only recommended after injuries and muscle tissue transplant, but also to treat chronic muscle loss, since the physical activity prevents the loss of muscle fibers, after small muscle contusions^5,36^. It is usually correlated with a proper nutrition. For example, protein intake can decrease the loss of muscle mass and supports physical therapy-related beneficial changes on tissue recovery^37^.

Physical therapy not only results in strengthening of unaffected muscles, but also promotes muscle regeneration and healing by releasing growth factors, stimulating vascularization, modulating the immune response and reducing scar formation^36,38,39^. Noteworthy, in small size muscle contusions/injuries research shows that immediate exercise is better than no effort and delayed effort for the contusion resolution. For example, in a randomized controlled animal model trial performed to determine the comparative rate of resolution of a contusion resulting from a mechanical trauma to the biceps femoris muscle, four exercise regimens were established: immediate and delayed (3 days after injury) running, and immediate and delayed (3 days after injury) swimming. Gregory et al. concluded that running is preferred over swimming and immediate onset is preferred to delayed (3 days after injury) onset for both running and swimming^36^. At the intracellular signaling level, it is worth mentioning that the physical exercise can upregulate the PI3K-Akt survival signaling pathway through Insulin Growth Factor 1 stimulation, thus preventing muscle atrophy^40^.

However, the use of physical therapy in VML is limited by the decreased ability of movement in the affected patient and by its relatively decreased ability of muscle regeneration and repair when used alone without surgery^5^.

### *3.3 Scaffolds and cell-based therapy*

Many biological scaffolds are now developed, and some are already tested in animal models. For muscle volume loss injuries, few of these biological scaffolds were already used in the clinic on human patients^7,41^. Biological scaffolds based on extracellular matrix (ECM), although not yet a current practice in surgical interventions and regenerative medicine for human patients, could promote the regeneration and repair in VML, in part by acting as regenerative templates and modulating healing processes, as shown in VML animal models^41^.

In one of the first studies involving innovative tissue engineering strategies for VML treatment in human patients, a surgical implantation of a 10 layered ECM-based biological scaffold derived from porcine intestinal submucosal in a 19-year-old patient with lost vastus medialis muscle due to an explosion, demonstrated marked gains in isokinetic performance after 4 months, in conjunction with physical therapy, although the muscle function remained significantly lower compared with the normal leg^42^. Moreover, as detailed in section 5, a recent clinical trial showed that an acellular biologic scaffold can be effective in functional tissue recovery and remodeling in patients with VML. The acellular biologic scaffold promotes the recruitment of myogenic progenitor cells, improves the innervation and formation of functional skeletal muscle^43^.**

Cell-based therapy using stem cells or other myogenic/non-myogenic cell types for skeletal muscle regeneration has been successful in animal models and showed promise in multiple clinical trials in humans^44^. These cells can promote the intrinsic capability of the injured muscle to regenerate and/or directly form new muscle fibers or other cellular constituents of the regenerated muscle. One major limitation of cell-based delivery is the significant cell death that may occur due to lack of engraftment, process called anoikis, which leads to the failure of the early clinical trials with myoblasts^44,45^. As detailed in section 5 and also observed by others^44^, a significant number of clinical trials using cell-based therapy are now ongoing or completed for the treatment of muscular dystrophies, such as Duchenne muscular dystrophy. Although some of these studies showed small or no difference in functional outcome^46^, others reported functional improvements^47^ and a significant increase not only in muscle volume, but also in contractile function of the muscle^44,48^.

Several limitations to the use of scaffolds and cell-based therapies exist. For example, the xenogeneic and allograft scaffolds and cells used in the injured muscle area have the risk of infectious disease transmission and the potential of generating an immune reaction from the patient^5^.**

### *3.4 Other strategies*

Other strategies can also be employed to stimulate muscle repair and regeneration. For example, in addition to a potential effect on reducing pain, acupuncture may be beneficial in muscle regeneration process. As an example, skeletal muscle proliferation and repair can be stimulated by the electrical acupuncture, in part by suppressing myostatin expression^49^*.* It is also believed that simulating exercise by promoting skeletal muscle contraction with low frequency electrical stimulation (Acu-LFES) combined with acupuncture prevents muscle loss and contributes to the muscle regeneration^5,50^.

## **4. Promising Tissue Engineering Treatments**

Tissue engineering involves three main components: the biomaterials (natural, synthetic), the cells (e.g stem cells) and chemical and physical factors (growth factors, electric field, mechanical stretch)^51^. In order to address the clinical problems that remain, while subsequently establishing novel strategies for muscle tissue engineering and regeneration, new technologies are being continuously investigated. One approach is by constructing complex muscle structures *in vitro* for subsequent implantation, as well as replacement of the missing muscle, while another method aims to develop tissue-like scaffolds which may be implanted to enhance new muscle formation from remaining tissue *in vivo*. Each with a differing focus – tissue engineering, and tissue regeneration, respectively – both of these methods mainly rely on the combination of scaffolds, cells, molecular and cellular interactions^5^. Materials designed to guide regeneration of skeletal muscle can be further classified as either acellular or cellular, with the latter incorporating myoblasts, stem cells or other musrelated cells into their matrix^1^.

### *4.1 Acellular and cellular scaffold-based strategies*

Biomaterials/scaffolds used in tissue engineering can be natural, synthetic materials and a combination of those, also named hybrid materials. The aim of scaffold-based strategies is to restore both the structure and function of the muscle, with minimal to no scarring formation^52^. Many *natural materials* have been employed, including collagen, fibrin, laminin, alginate, chitosan. Since collagen is one of the most important components of the muscle’s protective layers, such as the sarcolemma and endomysium, it was one of the main targets of investigation and has been tested in multiple studies^53^.

Although the natural materials are usually more biocompatible with the host after transplant and more bioactive, they can degrade more easily than synthetic materials, if not chemically modified. Biological scaffolds should have a long *in vivo* life to sustain muscle regeneration and be degraded while new skeletal muscle tissue is formed. Natural materials can be configured in different forms, which can present themselves as a film, sponge or hydrogel, and can be modified by cross-linking to improve their *in vivo* survival, cell attachment, mechanical characteristics and to evade the immune system of the host^54^.

*Synthetic scaffolds* are promising strategies for skeletal muscle tissue repair and regeneration. They present several advantages over the natural biomaterials, such as flexibility in chemical and physical modification, reproducibility in their preparation, modification and chemical properties. However, they usually tend to have a lower bioactivity than their natural counterparts. A wide array of strategies have been employed to generate scaffolds, including, but not limited to 3D printing, electrospinning, knitting, and a significant number of synthetic polymers, such as PLLA^55^, PDMS^56^, or PEG^57^ were developed, generally coated with adhesion peptides^51^.

*Hybrid scaffolds* are based on a combination of natural and synthetic materials, which can sometimes act in a synergistic manner, since they complement each other. The natural component is usually employed to stimulate the regeneration process by showing increased bioactivity, while the synthetic component brings in needed mechanical and physical properties^58^.

One of the major problems in VML injury is that the basal lamina at the injury site is damaged or lost, which negatively impacts the myogenesis process. Intact basal lamina can play the role of a regenerative template, also secreting chemotactic factors that recruit stem cells to the site of injury^26^ with an excellent outcome^1,59^. Decellularized extracellular matrix (dECM) scaffolds are a promising treatment, because they contain many of the biochemical cues needed for skeletal muscle repair and regeneration, such as cell adhesion, cell proliferation, and cell differentiation. Smoak et al. created a novel, high-throughput technique to fabricate dECM scaffolds with tunable physicochemical characteristics, while maintaining the structural matrix components required for regeneration^60^. A previously unknown strategy, Qiu et al. combined human umbilical cord mesenchymal stem cells with dECM scaffolds which regulated macrophage switch to the M2 phenotype and suppressed macrophage M1 phenotype, both of which are important in tissue regeneration process, as detailed in section 2^61^. Trevisan et al. proposed a diaphragm-derived extracellular matrix (ECM) as a scaffold for treatment of the congenital diaphragmatic hernia (CDH). Their team implanted diaphragmatic dECM-derived patches, which later demonstrated absence of rejection or hernia recurrence, as opposed to available treatments. Furthermore, Trevisan et al. concluded that their treatment was also able to promote generation of new blood vessels, new muscle fibers, and most importantly, to at least partially recover host diaphragmatic function. What is even more interesting, is that re-innervation was achieved by using Schwann cells in their mouse model. For the first time, this study showed that implantation of tissue-specific biologic scaffold can promote at least in part diaphragm muscle regeneration and overcome issues commonly observed for prosthetic materials^62^. In conclusion, Greg et al.’s study on dECM scaffold in combination with minced muscle for the treatment in the tibialis anterior of a VML model, asserts that although remaining muscle mass at site of injury is a source of myogenic cells and repair signals, a devitalized scaffold without myogenic cells is not sufficient to promote myogenesis in VML^63^.

Biological scaffolds in combination with different forms of muscle stimulation, such as exercise, enhance stem cell-based therapy in VML^64^ and may lead to a better outcome than either strategy (use of scaffolds and cell-based therapy) employed alone. Restoration of a pro-regeneration environment requires a multifactorial strategy, which may include combining scaffolds with the appropriate progenitor/stem cells. In animal studies, co-delivery of muscle progenitor cells in combination with ECM scaffolds has been shown to improve functional recovery following VML injury compared to ECM scaffold delivery alone^65^. Cellular co-delivery however, produces increased clinical complexity, in part because it requires surgical collection and expansion of a patient’s progenitor cells before implantation. One way to resolve this problem is through implantation of minced muscle (MM) autografts which contains satellite cells belonging to the stem cells niche. MM autografts can be obtained from normal skeletal muscle tissue and transferred into the injury site. The combination of a decellularized muscle scaffold with MM resulted in ~ 50% recovery of the muscle contractile force in a VML model. MM can increase myogenesis while simultaneously decreasing accumulation of collagen-enriched repair tissue^65^. Excessive collagen deposition by the fibroblasts can result in scar tissue formation, that can affect the regenerative process and the muscle functional performance after recovery^1^. A summary of several materials tested in different studies is presented in **Table 1**.

**Table 1:** Several Proposed Materials for Muscle Engineering^66-78^

Title	Comments	(Year), Reference
Ether-Oxygen Containing Electrospun Microfibrous and Sub-Microfibrous Scaffolds based on Poly(butylene 1,4-cyclohexanedicarboxylate)	Electrospun scaffolds improve cell adhesion, proliferation, and differentiation through cell alignment along fiber direction; allows for better cell infiltration and for oxygen and nutrient diffusion.	(2018), Ref. ^66^
Gelatin-genipin based biomaterials for skeletal muscle tissue engineering	Alternative myogenic stem cells, such as the adipose-derived, bone-marrow derived mesenchymal, perivascular, umbilical cord mesenchymal, induced pluripotent and embryonic stem cells, have myogenic potential and can be used in tissue engineering of skeletal muscle.	(2018), Ref. ^67^
Electroactive 3D scaffolds based on silk fibroin and water-borne polyaniline for skeletal muscle tissue engineering	Electroactive SF/PASA scaffolds with a suitable microenvironment, which can enhance myogenic differentiation of C2C12 cells, have a great potential for skeletal muscle regeneration.	(2017), Ref. ^68^
Natural polymeric hydrogel evaluation for skeletal muscle tissue engineering	Five commonly used natural polymeric materials were tested: collagen I, agarose, alginate, fibrin, and collagen chitosan; conclusion is that fibrin is the most suitable scaffold.	(2018), Ref. ^69^
Bio-inspired Hybrid Carbon Nanotube Muscles	Developed hybrid muscle powered by C2C12 skeletal muscle cells based on functionalized multi-walled carbon nanotubes sheets coated with poly ethylenedioxythyophene.	(2016), Ref. ^70^
Porous collagen scaffold, 3D	Development of a novel type of 3D porous collagen scaffold with concave microgrooves that mimic muscle basement membrane for skeletal muscle engineering.	(2015), Ref. ^53^
Covalently modified alginates with RGD (Arg-Gly-Asp)-containing cell adhesion ligands	Myoblast proliferation and differentiation could be regulated by varying the alginate monomeric ratio and the density of RGD ligands at the substrate surface; myoblast differentiation on these materials can be efficient at specific concentrations.	(2002), Ref. ^71^
Chitosan/alginate hydrogels	Chitosan/alginate (1/2) hydrogels provide a better environment for cell attachment and proliferation.	(2013), Ref. ^72^
Naturally derived and synthetic scaffolds	Biologically active and naturally derived materials (e.g. ECM); engineered synthetic polymers; naturally derived/synthetic “hybrid materials”.	(2014), Ref. ^73^
Biopolymer microthreads	Biopolymer microthreads are scaffolds promoting endogenous and exogenous tissue regeneration.	(2016), Ref. ^74^
Autologous minced muscle grafts	After 16 weeks postinjury, minced muscle graft transplantation promotes significant regeneration of innervated muscle fibers and reduces chronic injury in remaining muscle mass.	(2013), Ref. ^75^
Anisotropic Materials	Development of anisotropic scaffolds; this study examines how scaffold topographical, mechanical, and biochemical cues correlate to observed cellular function.	(2016), Ref. ^76^
Laminin-111 hydrogels	Hydrogel’s implantation showed significant improvements in muscle weights and heightened infiltration of stem, endothelial, hematopoietic, and immune cells at two weeks post-injury (murine model of muscle injury).	(2018), Ref. ^77^
Biomimetic sponges	Biomimetic sponges implanted in a mouse model of volumetric muscle loss supported stem , endothelial, and inflammatory cell infiltration; however, limited myofiber regeneration was observed at 2 weeks post-injury.	(2018), Ref. ^78^

### *4.2. Cell-based strategies*

Cell-based therapy using stem cells or other cell types for skeletal muscle regeneration has been successful in many animal models and multiple clinical trials in humans. Satellite cell is the most well characterized muscle stem cell, which is able to form new muscle fibers^5^. These cells can promote the intrinsic capability of the injured muscle to regenerate and/or directly form new muscle fibers or other cellular constituents of the regenerated muscle^44^. A significant number of clinical trials (ongoing or completed) are investigating the role of cell-therapy (e.g. stem cell-therapy) in muscle loss and other signs associated with muscular dystrophy, in particular in Duchenne muscular dystrophy, an X-linked recessive genetic pathology with progressive muscle loss and weakness. Although some of these studies showed small or no difference in the functional muscular outcome^46^, others reported functional improvements^47^ and a significant increase in muscle volume and contractile function of the muscle^48^, with no adverse effects of the autologous cell-based transplant on the host^44,47^. A summary of these clinical trials is presented in **Table 2**.

**Table 2:** Clinical Trials using Extracellular Matrix or Cell-based Therapy for Muscle Regeneration From ClinicalTrials.gov, a database of privately and publicly funded worldwide clinical studies; Interv. - Interventional; FDA – Food and Drug Administration; Dystr - Dystrophy; SCs – Stem Cells; EMG – Electromyography

Title of the Study	Study Number	Condition	Study Type	Comments/Results
Musculotendinous Tissue Repair Unit and Reinforcement (MTURR)	NCT01292876	Muscle Injury, Tendon Injury, Soft Tissue Injury	Interv.	A biological scaffold derived from animal derived collagen (FDA approved for “reinforcement of soft tissues repaired during tendon repair surgery") was used. 37.3% improvement (P<0.05) in strength, and 27.1% improvement in range-of-motion - 6 months43.
Clinical Study on Mesenchymal Stem Cells Used in the Reconstruction Surgery of the Supraspinatus Muscle Lesions	NCT03068988	Rotator Cuff Tear	Interv.	Mesenchymal stem cell-based therapy; stem cells transplant for supraspinatus muscle repair and regeneration after muscle injury; results unknown.
Stem Cell Therapy to Improve the Muscle Function of Patients With Partly Denervated Muscles of the Arm	NCT00755586	Brachial Plexus Injury	Interv.	Stem cell-based therapy: asses muscle improvement, muscle biopsies, quantitative needle EMG, muscle density analysis, force measurement, range of motion of the elbow joint and quality of life questionnaires; results unknown.
Allogeneic Adipose Derived Stem Cells for Werdnig Hoffman Patients	NCT02855112	Infantile Spinal Muscular Atrophy, I	Interv.	Adipose derived mesenchymal stem cell-based therapy; results unknown.
A Trial Comparing Three Orthobiologic Therapies on Atrophied Multifidus Muscles in Patients With Low Back Pain	NCT03618979	Atrophied multifidus muscles and axial lower back pain	Interv.	Autologous platelet rich plasma, platelet lysate and platelet poor plasma with an extracellular matrix injected directly into the atrophied multifidus muscle, in patients with axial lower back pain.
Trials investigating cell-therapy in Muscular Dystrophy	NCT03067831 NCT01834066 NCT02285673 NCT01610440 NCT02235844 NCT02484560 NCT01834040	Muscular Dystrophy	Interv.	Cell-based therapy in Muscular Dystrophy; only included completed, ongoing and recruiting clinical trials (withdrawn trials were not included);
Intramuscular Transplantation of Muscle Derived SCs and Adipose Derived Mesenchymal SCs-Facioscapulohumeral Dystr.	NCT02208713	Facioscapulohumeral Dystrophy	Interv.	Muscle-derived stem cell-based therapy and adipose-derived mesenchymal stem cell-based therapy; results unknown.

Noteworthy, combining biological scaffolds with cell-based therapy may lead to a better outcome than either strategy employed alone. For example, in a mouse model of VML the biological scaffolds combined with genetically-labelled muscle stem cells and other muscle associated cells showed improved muscle fiber and new blood vessel formation. Noteworthy, the innervation of the newly formed muscle is insufficient. However, both the muscle innervation and the function of the muscle are improved if the transplant is followed by physical therapy. Thus, a combination of biological scaffolds with cell-based therapy and exercise is a very promising strategy in VML (both acute and chronic)^64^.

There are multiple methods for combining biological scaffolds with cell-based therapy, such as electrospray^79^, microfluidics-based encapsulation^80^, micromolding, droplet/air and other methods. Each of these techniques have their own advantages and limitations^79-82^.**

Limitations of cell-based strategies are related to the most important steps of the transplantation process, from the autologous harvesting, the expansion, sorting and transplantation strategies, to the viability after transplantation^44^.

### *4.3 Other strategies*

Several other strategies, including skeletal muscle electrical stimulation, light therapy or heat stress are under investigation as means to stimulate or promote skeletal tissue recovery and regeneration, and/or prevent muscle loss. For example, intramuscular skeletal muscle stimulation promotes voluntary muscle activation after surgical repair^83^and it is useful in combating muscle disuse atrophy in humans, in correlation with proper nutrition, with the apport of proteins being a key factor. This is in part due to stimulation of protein synthesis in the skeletal muscle^84^.

Near-infrared light therapy is a non-invasive strategy shown to improve contractile function of an injured muscle during rehabilitation^85^, while low-level laser therapy was shown to have a positive effect on skeletal muscle morphology and volume in gastrocnemius muscle after burns^86^ and its combination with platelet rich plasma promoted better outcome for muscle regeneration after injuries compared to the use of either treatment alone, in a rat model^87^.

Moreover, heat stress can facilitate skeletal muscle regeneration in rat models, after a crush injury, process which is in part due to the heat-induced degradation process that may promote the regeneration and decrease the deposition of the collagen^88^. Temperature can modulate a wide range of intracellular molecules, such as heat shock proteins, that play an important role in the remodeling of skeletal muscle^89^.

### *4.4 Challenges and limitations*

#### *4.4.1 Limitations of biomaterials*

Biological scaffolds should have a long *in vivo* life to sustain muscle regeneration and be degraded while new skeletal muscle tissue is formed. To enable this, chemical crosslinking is employed, especially to natural polymers, which are easily degradable after *in vivo* transfer^54^. Another main problem in the use of various biological scaffolds, including the decellularized extracellular matrix-based materials, is the incomplete alignment of the regenerating tissue with the healthy one^1^. As suggested before, future studies may focus on the design of scaffolds resulting in useful degradation products, which contribute to the recruitment and/or support of stem cells for tissue regeneration and remodeling. Moreover, development of scaffolds that can help the differentiation of host stem cells or co-transplanted stem cells after *in vivo* transfer would be very useful^5^. Thus, a more comprehensive understanding of the potential of biological scaffolds, of the scaffold-host environment and scaffold-cell (co-transplanted or host cells) interactions in the skeletal smooth muscle regeneration is required.

#### *4.4.2 Limitations in the mouse models used*

Skeletal muscle cells are coated by basement membrane, which is a layer of extracellular matrix having an internal basal lamina directly linked to the plasma membrane of the cells and an external fibrillar reticular lamina. Many of the animal models of the skeletal muscle injury utilize methods of muscle injury that result in an intact basal lamina^90-92. ^This is not the case during VML, where the basal lamina is usually completely removed/destroyed. Intact basal lamina can play the role of a regenerative template, secreting chemotactic factors that recruit stem cells to the injury site^26^, with and an excellent outcome^1,59^. Thus, the methods using ischemia, toxins or crush-induced injuries^90-92^ may not be the best models to use for mimicking VML and uncovering new VML treatment strategies.

#### *4.4.3 Immune system, biomaterials and skeletal muscle regeneration*

Immune system plays a major role in skeletal muscle regeneration and the quality of the regenerated muscle in all three phases of natural muscle regeneration: inflammatory, repair and remodeling phases, as described in section 2. For example, one of the major events taking place within the repair phase is the activation of the stem-cell niche by the macrophages and other immune cells^3^. **

Allografts and xenografts transplanted biomaterials are sometimes rejected by the receiver's immune system. To prevent antigen recognition by the host’s immune system, these biomaterials are processed by chemical crosslinking or decellularization, which remove or cover the antigens^93^. The exact response of the immune system to various types of biological scaffolds and transplanted cells is not yet well known and future studies will provide potential clues on novel muscle tissue regeneration strategies.

#### *4.4.4 Lack of vascularization and/or innervation of the regenerated muscle*

Two of the main limitations when using scaffolds for tissue repair and regeneration are the lack or diminished formation of new vessels and innervation of the newly formed muscle. When scaffolds are transplanted at the site of injury, the lack of immediate blood supply is in part responsible for a potential failure^94^. The delayed in formation of new blood vessels, which may take up to 3-4 weeks to form, significantly impairs the process of repair and regeneration, may lead to cell death and scar formation^95^. A potential co-culture with endothelial cells^96^, stimulation with angiogenic factors^97^, together with integration of blood vessels within the scaffolds, which can be done via bioprinting or microfluidic methods^5^are potential ways of overcoming this problem.

During the process of regeneration, innervation (new nerves and endplates) for the newly formed muscle is required. This process involves the development of new neuromuscular junctions. Without proper innervation, any muscle will become atrophic^98^. Although *in vitro* studies show some promise^99^ it is unclear at this time how to stimulate the in vivo regeneration of new nerves and neuromuscular junction formation. Thus, further studies have to be designed for this purpose.

## **5. Towards a Human Host: Bioscaffolds and Cell-therapy in Clinical Trials**

Several clinical trials investigating the use of bioscaffolds and cell-based therapy in muscle pathologies are now ongoing or completed, with some of them showing promising outcomes.

For example, Dziki et al. performed a 13-patient cohort study on VML patients whose average muscle tissue loss was 66.2% to investigate a treatment plan that combined implantation of acellular bioscaffolds composed of mammalian extracellular ECM with early and aggressive physical therapy. Patients saw an improvement of 37.3% (P<0.05) in strength, and 27.1% improvement in range-of-motion tasks at six months following ECM implantation. Additionally, 7 of the 13 patients showed improvements compared to their per-surgical maximum within 6-8 weeks after surgery, as measured by strength testing. A sheet-like hyperechoic structure consistent with the ECM scaffold, overlying and adjacent to uninjured muscle was revealed by ultrasound imaging at one month post-surgery. In conclusion, in VML an acellular biologic scaffold can be effective in functional tissue recovery and remodeling. Acellular biologic scaffolds mediate their remodeling effects by promoting the recruitment of myogenic progenitor cells, improved innervation and functional skeletal muscle formation. These results highlight the benefit of using ECM bioscaffolds in VML therapy^43^ (see **Table 2**).

A significant number of clinical trials (ongoing or completed; **Table 2**) are investigating the role of cell-therapy (e.g. stem cell-therapy) in treating muscle loss and other signs associated with muscular dystrophy, in particular in Duchenne muscular dystrophy, an X-linked recessive genetic pathology with progressive muscle loss and weakness. Although some of these studies showed small or no difference in the functional outcome^46^, others reported functional improvements^47^ and a significant increase in muscle volume and contractile function of the muscle^48^, with no adverse effects of the autologous cell-based transplant^44,47^.

Noteworthy, most of these clinical trials, due to socio-economic implications, prevalence and other factors, are investigating muscular dystrophies, including the related muscle atrophy/loss, while for traumatic muscle loss there are only few ongoing clinical trials investigating the use of biological scaffolds/cell-based therapy, performed with patients affected by VML or muscle/nerve injury (see **Table 2**). Thus, there is an unmet clinical need for new clinical trials for traumatic muscle injury and for the development of new regenerative strategies in skeletal muscle pathologies.

## **6. Conclusion**

In severe muscle injuries with loss of muscle mass and function, such as VML, the mechanisms of the physiological muscle repair are overwhelmed. Thus, there is an unmet need for new skeletal muscle repair and regenerative strategies, such as the ones using biomimetic scaffolds and/or cell-based therapy, that can provide either structural or template support or regenerative signals to either transplanted or host cells. A wide range and types of biomaterials varying in structure, physical,chemical properties and function have been investigated. These strategies range from approaches that utilize biomaterials alone to those that combine materials with exogenous growth factors, and *ex vivo* cultured cells. Many of these strategies showed promise in structural and functional repair, regeneration and remodeling of the skeletal muscle in muscle loss injury and pathologies, in animal models and even in human patients, effects at least in part due to the recruitment of progenitor cells, improved innervation and formation of novel functional skeletal muscle.

A number of strategies using biomimetic scaffolds and/or cell-based therapies are in clinical trials for muscular dystrophy and VML, showing promising results. However, future approaches have to overcome the current limitations, by being significantly more efficient in muscle repair and regeneration. For this, a more comprehensive understanding of all the processes involved and how all components interact in skeletal muscle repair and regeneration is required. Future strategies must improve stem cell recruitment or survival/engraftment of the transplanted cells at the site of injury, their potential to regenerate muscle components and a functional muscle, blood vessels regeneration efficiency and restoration of muscle innervation.

## **KEY POINTS**

**◊** **Biomimetic scaffolds and/or cell-based therapy are promising strategies for skeletal muscle tissue repair and regeneration.**

**◊** **Several clinical trials using scaffolds and/or cell-based therapy for muscular dystrophy and VML are ongoing and completed, showing good results.**

**◊** **A more comprehensive understanding of all the processes involved and how all components interact in skeletal muscle repair and regeneration is required.**

**◊** **Future strategies must improve stem cell recruitment and survival, formation of functional muscle, blood vessels regeneration efficiency and restoration of muscle innervation.**

## References

[1] Grasman Jonathan M., Zayas Michelle J., Page Raymond L., Pins George D. (2015). Biomimetic scaffolds for regeneration of volumetric muscle loss in skeletal muscle injuries. Acta Biomaterialia.

[2] Yin Hang, Price Feodor, Rudnicki Michael A. (2013). Satellite Cells and the Muscle Stem Cell Niche. Physiological Reviews.

[3] Cittadella Vigodarzere Giorgio, Mantero Sara (2014). Skeletal muscle tissue engineering: strategies for volumetric constructs. Frontiers in Physiology.

[4] Patel Krishna H, Dunn Andrew J, Talovic Muhamed, Haas Gabriel J, Marcinczyk Madison, Elmashhady Hady, Kalaf Emily Growney, Sell Scott A, Garg Koyal (2019). Aligned nanofibers of decellularized muscle ECM support myogenic activity in primary satellite cells in vitro. Biomedical Materials.

[5] Liu Juan, Saul Dominik, Böker Kai Oliver, Ernst Jennifer, Lehman Wolfgang, Schilling Arndt F. (2018). Current Methods for Skeletal Muscle Tissue Repair and Regeneration. BioMed Research International.

[6] Corona Benjamin T., Rivera Jessica C., Owens Johnny G., Wenke Joseph C., Rathbone Christopher R. (2015). Volumetric muscle loss leads to permanent disability following extremity trauma. Journal of Rehabilitation Research and Development.

[7] Pollot Beth E., Corona Benjamin T. (2016). Volumetric Muscle Loss. Methods in Molecular Biology.

[8] Pansarasa O., Rossi D., Berardinelli A., Cereda C. (2013). Amyotrophic Lateral Sclerosis and Skeletal Muscle: An Update. Molecular Neurobiology.

[9] Jani-Acsadi Agnes, Ounpuu Sylvia, Pierz Kristan, Acsadi Gyula (2015). Pediatric Charcot-Marie-Tooth Disease. Pediatric Clinics of North America.

[10] Saure Carola, Caminiti Carolina, Weglinski Julieta, de Castro Perez Fernanda, Monges Soledad (2018). Energy expenditure, body composition, and prevalence of metabolic disorders in patients with Duchenne muscular dystrophy. Diabetes & Metabolic Syndrome: Clinical Research & Reviews.

[11] Kalyani Rita Rastogi, Corriere Mark, Ferrucci Luigi (2014). Age-related and disease-related muscle loss: the effect of diabetes, obesity, and other diseases. The Lancet Diabetes & Endocrinology.

[12] West Sarah L, Lok Charmaine E, Jamal Sophie A (2010). Fracture Risk Assessment in Chronic Kidney Disease, Prospective Testing Under Real World Environments (FRACTURE): a prospective study. BMC Nephrology.

[13] Järvinen Tero A. H., Järvinen Teppo L. N., Kääriäinen Minna, Kalimo Hannu, Järvinen Markku (2005). Muscle Injuries. The American Journal of Sports Medicine.

[14] Grogan Brian F., Hsu Joseph R. (2011). Volumetric Muscle Loss. American Academy of Orthopaedic Surgeon.

[15] Bianchi Bernardo, Copelli Chiara, Ferrari Silvano, Ferri Andrea, Sesenna Enrico (2009). Free flaps: Outcomes and complications in head and neck reconstructions. Journal of Cranio-Maxillofacial Surgery.

[16] Turner Neill J., Badylak Stephen F. (2011). Regeneration of skeletal muscle. Cell and Tissue Research.

[17] Bodine-Fowler Sue (1994). Skeletal muscle regeneration after injury: An overview. Journal of Voice.

[18] Kimura Naoki, Hirata Shinya, Miyasaka Nobuyuki, Kawahata Kimito, Kohsaka Hitoshi (2015). Injury and Subsequent Regeneration of Muscles for Activation of Local Innate Immunity to Facilitate the Development and Relapse of Autoimmune Myositis in C57BL/6 Mice. Arthritis & Rheumatology.

[19] Tidball James G., Villalta S. Armando (2010). Regulatory interactions between muscle and the immune system during muscle regeneration. American Journal of Physiology-Regulatory, Integrative and Comparative Physiology.

[20] Tidball James G. (2011). Mechanisms of Muscle Injury, Repair, and Regeneration. Comprehensive Physiology.

[21] Deng Bo, Wehling-Henricks Michelle, Villalta S. Armando, Wang Ying, Tidball James G. (2012). IL-10 Triggers Changes in Macrophage Phenotype That Promote Muscle Growth and Regeneration. The Journal of Immunology.

[22] Villalta S. A., Rinaldi C., Deng B., Liu G., Fedor B., Tidball J. G. (2010). Interleukin-10 reduces the pathology of mdx muscular dystrophy by deactivating M1 macrophages and modulating macrophage phenotype. Human Molecular Genetics.

[23] Villalta S. Armando, Deng Bo, Rinaldi Chiara, Wehling-Henricks Michelle, Tidball James G. (2011). IFN-γ Promotes Muscle Damage in the mdx Mouse Model of Duchenne Muscular Dystrophy by Suppressing M2 Macrophage Activation and Inhibiting Muscle Cell Proliferation. The Journal of Immunology.

[24] Pettersson Folke, Malker Birgitta (1989). Invasive carcinoma of the uterine cervix following diagnosis and treatment of in situ carcinoma. Record linkage study within a National Cancer Registry. Radiotherapy and Oncology.

[25] HUARD JOHNNY, LI YONG, FU FREDDIE H. (2002). MUSCLE INJURIES AND REPAIR. The Journal of Bone and Joint Surgery-American Volume.

[26] Hayashi Shinichiro, Aso Hisashi, Watanabe Kouichi, Nara Hidetoshi, Rose Michael T., Ohwada Shyuichi, Yamaguchi Takahiro (2000). Sequence of IGF-I, IGF-II, and HGF expression in regenerating skeletal muscle. Histochemistry and Cell Biology.

[27] Aguilar Carlos A, Greising Sarah M, Watts Alain, Goldman Stephen M, Peragallo Chelsea, Zook Christina, Larouche Jacqueline, Corona Benjamin T (2018). Multiscale analysis of a regenerative therapy for treatment of volumetric muscle loss injury.. Cell death discovery.

[28] Whittington B Understanding the 3 Phases of Muscle Healing. Athletico physical therapy. Accessed on March 25th, 2019.

[29] Eckardt André, Fokas Konstantinos (2003). Microsurgical reconstruction in the head and neck region: an 18-year experience with 500 consecutive cases. Journal of Cranio-Maxillofacial Surgery.

[30] Stevanovic Milan V., Cuéllar Vanessa G., Ghiassi Alidad, Sharpe Frances (2016). Single-stage Reconstruction of Elbow Flexion Associated with Massive Soft-Tissue Defect Using the Latissimus Dorsi Muscle Bipolar Rotational Transfer. Plastic and Reconstructive Surgery - Global Open.

[31] Barrera-Ochoa Sergi, Collado-Delfa Jose Manuel, Sallent Andrea, Lluch Alejandro, Velez Roberto (2017). Free Neurovascular Latissimus Dorsi Muscle Transplantation for Reconstruction of Hip Abductors. Plastic and Reconstructive Surgery - Global Open.

[32] Maldonado Andrés A., Kircher Michelle F., Spinner Robert J., Bishop Allen T., Shin Alexander Y. (2017). Free Functioning Gracilis Muscle Transfer With and Without Simultaneous Intercostal Nerve Transfer to Musculocutaneous Nerve for Restoration of Elbow Flexion After Traumatic Adult Brachial Pan-Plexus Injury. The Journal of Hand Surgery.

[33] Sue Gloria R., Ho Oscar H. (2019). Microsurgical Reconstruction of the Smile. Annals of Plastic Surgery.

[34] Lin BY, Guo QF, Liu YY, Huang K, Zhang C, Shen LF. (2018). [Transfer of gastrocnemius muscle flap for postoperative infection with patellar internal fixation]. Zhongguo Gu Shang (in Chinese).

[35] Wang W, Xu B, Zhu J, Yang C, Shen S, Qian Y (2018). Maxillary reconstruction using rectus femoris muscle flap and sagittal 
mandibular ramus/coronoid process graft pedicled with temporalis muscle. Medicina Oral Patología Oral y Cirugia Bucal.

[36] Gregory T M, Heckmann R A, Francis R S (1995). The effect of exercise on the presence of leukocytes, erythrocytes and collagen fibers in skeletal muscle after contusion.. Journal of manipulative and physiological therapeutics.

[37] Sundström-Rehal Martin, Tardif Nicolas, Rooyackers Olav (2019). Can exercise and nutrition stimulate muscle protein gain in the ICU patient?. Current Opinion in Clinical Nutrition and Metabolic Care.

[38] Brutsaert Tom D, Gavin Timothy P, Fu Zhenxing, Breen Ellen C, Tang Kechun, Mathieu-Costello Odile, Wagner Peter D (2002). Regional differences in expression of VEGF mRNA in rat gastrocnemius following 1 hr exercise or electrical stimulation.. BMC physiology.

[39] Faria F E T, Ferrari R J, Distefano G, Ducatti A C, Soares K F, Montebelo M I L, Minamoto V B (2008). The onset and duration of mobilization affect the regeneration in the rat muscle.. Histology and histopathology.

[40] Chen Y., Sood S., Biada J., Roth R., Rabkin R. (2008). Increased workload fully activates the blunted IRS-1/PI3-kinase/Akt signaling pathway in atrophied uremic muscle. Kidney International.

[41] Sicari B. M., Rubin J. P., Dearth C. L., Wolf M. T., Ambrosio F., Boninger M., Turner N. J., Weber D. J., Simpson T. W., Wyse A., Brown E. H. P., Dziki J. L., Fisher L. E., Brown S., Badylak S. F. (2014). An Acellular Biologic Scaffold Promotes Skeletal Muscle Formation in Mice and Humans with Volumetric Muscle Loss. Science Translational Medicine.

[42] Mase Vincent J., Hsu Joseph R., Wolf Steven E., Wenke Joseph C., Baer David G., Owens Johnny, Badylak Stephen F., Walters Thomas J. (2010). Clinical Application of an Acellular Biologic Scaffold for Surgical Repair of a Large, Traumatic Quadriceps Femoris Muscle Defect. Orthopedics.

[43] Dziki Jenna, Badylak Stephen, Yabroudi Mohammad, Sicari Brian, Ambrosio Fabrisia, Stearns Kristen, Turner Neill, Wyse Aaron, Boninger Michael L, Brown Elke H P, Rubin J Peter (2016). An acellular biologic scaffold treatment for volumetric muscle loss: results of a 13-patient cohort study. npj Regenerative Medicine.

[44] Qazi Taimoor H., Duda Georg N., Ort Melanie J., Perka Carsten, Geissler Sven, Winkler Tobias (2019). Cell therapy to improve regeneration of skeletal muscle injuries. Journal of Cachexia, Sarcopenia and Muscle.

[45] Huard J, Roy R, Bouchard J P, Malouin F, Richards C L, Tremblay J P (1992). Human myoblast transplantation between immunohistocompatible donors and recipients produces immune reactions.. Transplantation proceedings.

[46] Sarveazad Arash, Newstead Graham L., Mirzaei Rezvan, Joghataei Mohammad Taghi, Bakhtiari Mehrdad, Babahajian Asrin, Mahjoubi Bahar (2017). A new method for treating fecal incontinence by implanting stem cells derived from human adipose tissue: preliminary findings of a randomized double-blind clinical trial. Stem Cell Research & Therapy.

[47] Peters Kenneth M., Dmochowski Roger R., Carr Lesley K., Robert Magali, Kaufman Melissa R., Sirls Larry T., Herschorn Sender, Birch Colin, Kultgen Patricia L., Chancellor Michael B. (2014). Autologous Muscle Derived Cells for Treatment of Stress Urinary Incontinence in Women. Journal of Urology.

[48] Winkler Tobias, Perka Carsten, von Roth Philipp, Agres Alison N., Plage Henning, Preininger Bernd, Pumberger Matthias, Geissler Sven, Hagai Esther Lukasiewicz, Ofir Racheli, Pinzur Lena, Eyal Eli, Stoltenburg-Didinger Gisela, Meisel Christian, Consentius Christine, Streitz Mathias, Reinke Petra, Duda Georg N., Volk Hans-Dieter (2018). Immunomodulatory placental-expanded, mesenchymal stromal cells improve muscle function following hip arthroplasty. Journal of Cachexia, Sarcopenia and Muscle.

[49] Takaoka Yutaka, Ohta Mika, Ito Akihiko, Takamatsu Kunihiko, Sugano Aki, Funakoshi Kotaro, Takaoka Nobuo, Sato Nobuko, Yokozaki Hiroshi, Arizono Naoki, Goto Shuji, Maeda Eiichi (2007). Electroacupuncture suppresses myostatin gene expression: cell proliferative reaction in mouse skeletal muscle. Physiological Genomics.

[50] Su Zhen, Robinson Alayna, Hu Li, Klein Janet D., Hassounah Faten, Li Min, Wang Haidong, Cai Hui, Wang Xiaonan H. (2015). Acupuncture plus Low-Frequency Electrical Stimulation (Acu-LFES) Attenuates Diabetic Myopathy by Enhancing Muscle Regeneration. PLOS ONE.

[51] Beldjilali-Labro Megane, Garcia Garcia Alejandro, Farhat Firas, Bedoui Fahmi, Grosset Jean-François, Dufresne Murielle, Legallais Cécile (2018). Biomaterials in Tendon and Skeletal Muscle Tissue Engineering: Current Trends and Challenges. Materials.

[52] Grefte Sander, Kuijpers-Jagtman Anne Marie, Torensma Ruurd, Von den Hoff Johannes W. (2007). Skeletal Muscle Development and Regeneration. Stem Cells and Development.

[53] Chen Shangwu, Nakamoto Tomoko, Kawazoe Naoki, Chen Guoping (2015). Engineering multi-layered skeletal muscle tissue by using 3D microgrooved collagen scaffolds. Biomaterials.

[54] Chaudhari Atul, Vig Komal, Baganizi Dieudonné, Sahu Rajnish, Dixit Saurabh, Dennis Vida, Singh Shree, Pillai Shreekumar (2016). Future Prospects for Scaffolding Methods and Biomaterials in Skin Tissue Engineering: A Review. International Journal of Molecular Sciences.

[55] Altomare L., Gadegaard N., Visai L., Tanzi M.C., Farè S. (2010). Biodegradable microgrooved polymeric surfaces obtained by photolithography for skeletal muscle cell orientation and myotube development. Acta Biomaterialia.

[56] Lam Mai T., Sim Sylvie, Zhu Xiaoyue, Takayama Shuichi (2006). The effect of continuous wavy micropatterns on silicone substrates on the alignment of skeletal muscle myoblasts and myotubes. Biomaterials.

[57] Wang Ling, Wu Yaobin, Guo Baolin, Ma Peter X. (2015). Nanofiber Yarn/Hydrogel Core–Shell Scaffolds Mimicking Native Skeletal Muscle Tissue for Guiding 3D Myoblast Alignment, Elongation, and Differentiation. ACS Nano.

[58] Mallick Kajal K (2013). Biomaterial scaffolds for tissue engineering. Frontiers in Bioscience.

[59] Sanes Joshua R. (2003). The Basement Membrane/Basal Lamina of Skeletal Muscle. Journal of Biological Chemistry.

[60] Smoak Mollie M., Han Albert, Watson Emma, Kishan Alysha, Grande-Allen K. Jane, Cosgriff-Hernandez Elizabeth, Mikos Antonios G. (2019). Fabrication and Characterization of Electrospun Decellularized Muscle-Derived Scaffolds. Tissue Engineering Part C: Methods.

[61] Qiu Xinyu, Liu Shiyu, Zhang Hao, Zhu Bin, Su Yuting, Zheng Chenxi, Tian Rong, Wang Miao, Kuang Huijuan, Zhao Xinyi, Jin Yan (2018). Mesenchymal stem cells and extracellular matrix scaffold promote muscle regeneration by synergistically regulating macrophage polarization toward the M2 phenotype. Stem Cell Research & Therapy.

[62] Trevisan Caterina, Maghin Edoardo, Dedja Arben, Caccin Paola, de Cesare Niccolò, Franzin Chiara, Boso Daniele, Pesce Paola, Caicci Federico, Boldrin Francesco, Urbani Luca, De Coppi Paolo, Pozzobon Michela, Pavan Piero, Piccoli Martina (2019). Allogenic tissue-specific decellularized scaffolds promote long-term muscle innervation and functional recovery in a surgical diaphragmatic hernia model. Acta Biomaterialia.

[63] Garg Koyal, Ward Catherine L., Rathbone Christopher R., Corona Benjamin T. (2014). Transplantation of devitalized muscle scaffolds is insufficient for appreciable de novo muscle fiber regeneration after volumetric muscle loss injury. Cell and Tissue Research.

[64] Quarta Marco, Cromie Melinda, Chacon Robert, Blonigan Justin, Garcia Victor, Akimenko Igor, Hamer Mark, Paine Patrick, Stok Merel, Shrager Joseph B., Rando Thomas A. (2017). Bioengineered constructs combined with exercise enhance stem cell-mediated treatment of volumetric muscle loss. Nature Communications.

[65] Kasukonis Benjamin, Kim John, Brown Lemuel, Jones Jake, Ahmadi Shahryar, Washington Tyrone, Wolchok Jeffrey (2016). Codelivery of Infusion Decellularized Skeletal Muscle with Minced Muscle Autografts Improved Recovery from Volumetric Muscle Loss Injury in a Rat Model. Tissue Engineering Part A.

[66] Bloise Nora, Berardi Emanuele, Gualandi Chiara, Zaghi Elisa, Gigli Matteo, Duelen Robin, Ceccarelli Gabriele, Cortesi Emanuela, Costamagna Domiziana, Bruni Giovanna, Lotti Nadia, Focarete Maria, Visai Livia, Sampaolesi Maurilio (2018). Ether-Oxygen Containing Electrospun Microfibrous and Sub-Microfibrous Scaffolds Based on Poly(butylene 1,4-cyclohexanedicarboxylate) for Skeletal Muscle Tissue Engineering. International Journal of Molecular Sciences.

[67] Pantelic Molly N., Larkin Lisa M. (2018). Stem Cells for Skeletal Muscle Tissue Engineering. Tissue Engineering Part B: Reviews.

[68] Zhang Mengyao, Guo Baolin (2017). Electroactive 3D Scaffolds Based on Silk Fibroin and Water-Borne Polyaniline for Skeletal Muscle Tissue Engineering. Macromolecular Bioscience.

[69] Pollot Beth E., Rathbone Christopher R., Wenke Joseph C., Guda Teja (2017). Natural polymeric hydrogel evaluation for skeletal muscle tissue engineering. Journal of Biomedical Materials Research Part B: Applied Biomaterials.

[70] Kim Tae Hyeob, Kwon Cheong Hoon, Lee Changsun, An Jieun, Phuong Tam Thi Thanh, Park Sun Hwa, Lima Márcio D., Baughman Ray H., Kang Tong Mook, Kim Seon Jeong (2016). Bio-inspired Hybrid Carbon Nanotube Muscles. Scientific Reports.

[71] Rowley Jon A., Mooney David J. (2002). Alginate type and RGD density control myoblast phenotype. Journal of Biomedical Materials Research.

[72] Baysal Kemal, Aroguz Ayse Z., Adiguzel Zelal, Baysal Bahattin M. (2013). Chitosan/alginate crosslinked hydrogels: Preparation, characterization and application for cell growth purposes. International Journal of Biological Macromolecules.

[73] Wolf Matthew T., Dearth Christopher L., Sonnenberg Sonya B., Loboa Elizabeth G., Badylak Stephen F. (2015). Naturally derived and synthetic scaffolds for skeletal muscle reconstruction. Advanced Drug Delivery Reviews.

[74] O’Brien Megan P., Carnes Meagan E., Page Raymond L., Gaudette Glenn R., Pins George D. (2016). Designing Biopolymer Microthreads for Tissue Engineering and Regenerative Medicine. Current Stem Cell Reports.

[75] Corona B. T., Garg K., Ward C. L., McDaniel J. S., Walters T. J., Rathbone C. R. (2013). Autologous minced muscle grafts: a tissue engineering therapy for the volumetric loss of skeletal muscle. American Journal of Physiology-Cell Physiology.

[76] Jana Soumen, Levengood Sheeny K. Lan, Zhang Miqin (2016). Anisotropic Materials for Skeletal-Muscle-Tissue Engineering. Advanced Materials.

[77] Marcinczyk Madison, Dunn Andrew, Haas Gabriel, Madsen Josh, Scheidt Robert, Patel Krishna, Talovic Muhamed, Garg Koyal (2019). The Effect of Laminin-111 Hydrogels on Muscle Regeneration in a Murine Model of Injury. Tissue Engineering Part A.

[78] Haas Gabriel J., Dunn Andrew J., Marcinczyk Madison, Talovic Muhamed, Schwartz Mark, Scheidt Robert, Patel Anjali D., Hixon Katherine R., Elmashhady Hady, McBride-Gagyi Sarah H., Sell Scott A., Garg Koyal (2018). Biomimetic sponges for regeneration of skeletal muscle following trauma. Journal of Biomedical Materials Research Part A.

[79] Vossoughi Amin, Matthew Howard W. T. (2018). Encapsulation of mesenchymal stem cells in glycosaminoglycans-chitosan polyelectrolyte microcapsules using electrospraying technique: Investigating capsule morphology and cell viability. Bioengineering & Translational Medicine.

[80] Feng Huanhuan, Zheng Tingting, Li Mingyu, Wu Junwei, Ji Hongjun, Zhang Jiaheng, Zhao Weiwei, Guo Jinhong (2019). Droplet‐based microfluidics systems in biomedical applications. ELECTROPHORESIS.

[81] Tiruvannamalai-Annamalai Ramkumar, Armant David Randall, Matthew Howard W. T. (2014). A Glycosaminoglycan Based, Modular Tissue Scaffold System for Rapid Assembly of Perfusable, High Cell Density, Engineered Tissues. PLoS ONE.

[82] Annamalai Ramkumar T., Matthew Howard W. T. (2019). Transport Analysis of Engineered Liver Tissue Fabricated Using a Capsule-Based, Modular Approach. Annals of Biomedical Engineering.

[83] Hollis Sharon, McClure Philip (2017). Intramuscular Electrical Stimulation for Muscle Activation of the Tibialis Anterior After Surgical Repair: A Case Report. Journal of Orthopaedic & Sports Physical Therapy.

[84] Wall Benjamin T., Morton James P., van Loon Luc J. C. (2014). Strategies to maintain skeletal muscle mass in the injured athlete: Nutritional considerations and exercise mimetics. European Journal of Sport Science.

[85] Larkin-Kaiser Kelly A., Christou Evangelos, Tillman Mark, George Steven, Borsa Paul A. (2015). Near-Infrared Light Therapy to Attenuate Strength Loss After Strenuous Resistance Exercise. Journal of Athletic Training.

[86] Martins Francielle, Rennó Ana Cláudia Muniz, Oliveira Flávia de, Minatel Natália Peruchi, Bortolin Jeferson André, Quintana Hananiah Tardivo, Aveiro Mariana Chaves (2015). Low-level laser therapy modulates musculoskeletal loss in a skin burn model in rats. Acta Cirurgica Brasileira.

[87] Garcia Thiago Alves, Camargo Regina Celi Trindade, Koike Tatiana Emy, Ozaki Guilherme Akio Tamura, Castoldi Robson Chacon, Camargo Filho José Carlos Silva (2017). Histological analysis of the association of low level laser therapy and platelet-rich plasma in regeneration of muscle injury in rats. Brazilian Journal of Physical Therapy.

[88] Takeuchi Kousuke, Hatade Takuya, Wakamiya Soushi, Fujita Naoto, Arakawa Takamitsu, Miki Akinori (2014). Heat stress promotes skeletal muscle regeneration after crush injury in rats. Acta Histochemica.

[89] Obi Syotaro, Nakajima Toshiaki, Hasegawa Takaaki, Nakamura Fumitaka, Sakuma Masashi, Toyoda Shigeru, Tei Chuwa, Inoue Teruo (2018). Heat induces myogenic transcription factors of myoblast cells via transient receptor potential vanilloid 1 (Trpv1). FEBS Open Bio.

[90] Borselli Cristina, Storrie Hannah, Benesch-Lee Frank, Shvartsman Dmitry, Cezar Christine, Lichtman Jeff W., Vandenburgh Herman H., Mooney David J. (2009). Functional muscle regeneration with combined delivery of angiogenesis and myogenesis factors. Proceedings of the National Academy of Sciences.

[91] Wang Lin, Cao Lan, Shansky Janet, Wang Zheng, Mooney David, Vandenburgh Herman (2014). Minimally Invasive Approach to the Repair of Injured Skeletal Muscle With a Shape-memory Scaffold. Molecular Therapy.

[92] Stratos Ioannis, Graff Johannes, Rotter Robert, Mittlmeier Thomas, Vollmar Brigitte (2010). Open blunt crush injury of different severity determines nature and extent of local tissue regeneration and repair. Journal of Orthopaedic Research.

[93] GILBERT T, SELLARO T, BADYLAK S (2006). Decellularization of tissues and organs. Biomaterials.

[94] Jin Yan, Bi Huanjing (2013). Current progress of skin tissue engineering: Seed cells, bioscaffolds, and construction strategies. Burns & Trauma.

[95] Nyame Theodore T., Chiang H. Abraham, Leavitt Tripp, Ozambela Manuel, Orgill Dennis P. (2015). Tissue-Engineered Skin Substitutes. Plastic and Reconstructive Surgery.

[96] Haro Hirotaka, Kato Tsuyoshi, Komori Hiromichi, Osada Motonobu, Shinomiya Kenichi (2002). Vascular endothelial growth factor (VEGF)-induced angiogenesis in herniated disc resorption. Journal of Orthopaedic Research.

[97] Thomopoulos Stavros, Kim H Mike, Das Rosalina, Silva Matthew J, Sakiyama-Elbert Shelly, Amiel David, Gelberman Richard H (2010). The Effects of Exogenous Basic Fibroblast Growth Factor on Intrasynovial Flexor Tendon Healing in a Canine Model. The Journal of Bone and Joint Surgery-American Volume.

[98] Järvinen Tero A.H., Järvinen Teppo L.N., Kääriäinen Minna, Äärimaa Ville, Vaittinen Samuli, Kalimo Hannu, Järvinen Markku (2007). Muscle injuries: optimising recovery. Best Practice & Research Clinical Rheumatology.

[99] Larkin Lisa M, Van der Meulen Jack H, Dennis Robert G, Kennedy Jeffrey B (2006). Functional evaluation of nerve-skeletal muscle constructs engineered in vitro.. In vitro cellular & developmental biology. Animal.

